# Development of a Community-Based Rehabilitation Intervention for People with Schizophrenia in Ethiopia

**DOI:** 10.1371/journal.pone.0143572

**Published:** 2015-11-30

**Authors:** Laura Asher, Abebaw Fekadu, Charlotte Hanlon, Gemechu Mideksa, Julian Eaton, Vikram Patel, Mary J. De Silva

**Affiliations:** 1 Centre for Global Mental Health, London School of Hygiene and Tropical Medicine, London, United Kingdom; 2 Department of Psychiatry, School of Medicine, College of Health Sciences, Addis Ababa University, Addis Ababa, Ethiopia; 3 Centre for Affective Disorders, Department of Psychological Medicine, Institute of Psychiatry, King’s College London, London, United Kingdom; 4 Center for Global Mental Health, Institute of Psychiatry, King’s College London, London, United Kingdom; 5 RAPID (Rehabilitation And Prevention Initiative against Disabilities) CBR Project, Adama, Ethiopia; 6 CBM West Africa Regional Office, Lome, Togo; 7 Sangath, Goa, India; Benito Menni Complejo Asistencial en Salud Mental, SPAIN

## Abstract

**Background:**

Community-based rehabilitation (CBR) is a multi-sectoral strategy to improve the functioning and quality of life of people with disabilities. The RISE (Rehabilitation Intervention for people with Schizophrenia in Ethiopia) trial will evaluate the effectiveness of CBR for people with schizophrenia in Ethiopia. Nevertheless, the components of CBR that are both feasible and likely to prove effective in low and middle-income countries such as Ethiopia are unclear.

**Methods:**

In this study intervention development work was undertaken to design a CBR intervention that is acceptable and feasible in the local context. The development work consisted of five phases. 1: Identify potential components of CBR for schizophrenia, 2: Situational analysis, 3: Determine feasibility of CBR (Theory of Change workshops with experts and local stakeholders), 4: Determine acceptability of CBR (16 in-depth interviews and five focus group discussions with people with schizophrenia, caregivers, health workers and community leaders) and 5: Synthesise results to finalise intervention. A Theory of Change map was constructed showing the causal pathway for how we expect CBR to achieve its impact.

**Results:**

People with schizophrenia in rural Ethiopia experience family conflict, difficulty participating in work and community life, and stigma. Stakeholders perceived CBR to be acceptable and useful to address these problems. The focus of CBR will be on the individual developing the skills and confidence to perform their previous or desired roles and activities. To ensure feasibility, non-health professionals will be trained to deliver CBR and provide supervision, rather than mental health specialists. Novel components of CBR for schizophrenia included family intervention and dealing with distressing symptoms. Microfinance was excluded due to concerns about stress and exploitation. Community mobilisation was viewed as essential to ensure the effectiveness and sustainability of CBR.

**Conclusion:**

Extensive formative research using a variety of methods has enabled the design of a culturally appropriate CBR intervention for people with schizophrenia that is acceptable and feasible.

## Introduction

Many people with schizophrenia experience severe and chronic illness; in Ethiopia 38% had episodic symptoms and 19% had continuous symptoms over a 10-year period whilst 11.8% had complete remission after one episode [1]. Reflecting global patterns, in Ethiopia, people with schizophrenia have high levels of disability [2], family burden [3], stigma [4, 5], and mortality [6]. Despite this, the majority of people with schizophrenia in low and middle income countries (LMIC) do not have access to adequate care; in Ethiopia, the treatment gap is 90% [7]. Human rights violations also occur [8] and many are the victims of violence [9]. Treatment with antipsychotic medication alone is often not adequate to achieve functional recovery and social reintegration [11]. Psychosocial or psychiatric rehabilitation is recognised globally as an essential component of care for people with schizophrenia. Psychosocial rehabilitation is “a process that facilitates the opportunity for individuals…to reach their optimum level of independent functioning in the community”[12]. There is also increasing agreement that the management of schizophrenia should be guided by the principle of recovery, in which the focus is on empowerment, self-direction, personal responsibility and hope [13, 14]. The WHO’s mental health Gap Action Programme (mhGAP) recommends that schizophrenia management should include psychosocial interventions, including community-based rehabilitation (CBR), where available, although evidence from LMIC settings is limited [15, 16]. CBR is a strategy that aims to reduce disability and improve the quality of life and social inclusion of people with disabilities. CBR echoes the ethos of psychosocial rehabilitation, particularly drawing on recovery values, whilst reflecting the particular needs of low-income settings [17].

Programmes cover one or more of the CBR pillars (health, education, livelihoods, social and empowerment), focused on facilitating individuals to access existing resources [17]. CBR is put into practice through the joint endeavours of people with disabilities, their caregivers, community members and public sector services e.g. health services [18]. There is a long-standing tradition of CBR and a global network of CBR programmes, but these have historically focused on other disabilities. There is now increasing recognition that people with mental illnesses may receive substantial benefit from CBR. As such mental health has been incorporated into CBR programmes in Sri Lanka, India, West Africa, China and Latin America [17, 19–21]. A systematic review found that aspects of CBR may improve clinical outcomes and functioning for schizophrenia, dementia and intellectual disabilities in LMICs [18]. There is evidence from randomised controlled trials (RCTs) to support the effectiveness of assertive community treatment (ACT) for people with schizophrenia in South Africa [22] and psycho-educational family interventions in China [23, 24]. However no RCTs of holistic packages of CBR for schizophrenia that involved community mobilisation (defined as “a strategy which aims to engage community members and empower them for change or action”[17]) or that focused primarily on any pillar other than health, were included [18]. The more recent COmmunity care for People with Schizophrenia in India (COPSI) trial [25] found collaborative community care modestly improved disability and symptoms in people with schizophrenia [26]. Whilst influenced by CBR, the intervention did not include community mobilisation.

In summary, CBR is a promising intervention for people with chronic and disabling schizophrenia (due to illness factors or structural factors which lead to drop out from care) particularly in low-income settings where treatment options for this group are limited. Yet there has been no systematic adaptation of comprehensive CBR for people with schizophrenia nor assessment of its effectiveness in low and middle-income countries. The Rehabilitation Intervention for people with Schizophrenia in Ethiopia (RISE) project aims to adapt CBR for people with schizophrenia in a rural Ethiopian setting and to assess its effectiveness. The RISE trial (NCT02160249) will determine whether CBR as an adjunct to facility-based care is superior to facility-based care alone in reducing disability in people with schizophrenia who remain symptomatic or disabled after six months of treatment. This will be the first comprehensive CBR programme for mental illness to be evaluated in a clinical trial in Africa. The intensive CBR intervention will be targeted at those with the greatest need. The rationale for this is (i) the aim of CBR is to reduce disability, so it is appropriate primarily for those who are disabled and (ii) to increase feasibility for scale-up by restricting the intervention to those most in need. RISE is nested in PRIME (Programme for Improving Mental healthcarE), a five-country research consortium, including Ethiopia, which aims to generate evidence on the integration of mental health into primary care in LMIC [27, 28]. As part of PRIME, primary care staff in Sodo district, Ethiopia, have been trained to deliver packages of care for people with schizophrenia including prescription of antipsychotic medication, follow-up, limited adherence support, basic psychoeducation and community awareness-raising [29]. Psychoeducation refers to the education of people with mental illness to increase their knowledge and understanding of their treatment and illness. This facility based care constitutes treatment as usual in the control arm of the RISE trial, and will be delivered in conjunction with CBR in the intervention arm.

The importance of intervention development prior to testing complex mental health interventions is widely acknowledged, particularly in LMIC [30–33]. This work helps to design an intervention that is acceptable and feasible to both its recipients and those delivering it, and is ultimately more effective. The particular benefits include:

- Ensuring the intervention is appropriate to local resources and health services structures [32, 34].
- Identifying contextually-mediated barriers to delivery [33].
- Ensuring the cross-cultural applicability and relevance [30].
- Getting buy-in from national and local stakeholders [30].

This paper summarises the formative research used to design an acceptable, feasible and sustainable CBR intervention that is likely to be effective for people with schizophrenia in Ethiopia, to be tested in a pilot and subsequently in the RISE trial. At the outset we acknowledged two key challenges. First, how to develop a CBR intervention in a setting with few public sector mental health resources. Second, how to strike the balance between an intervention which is likely to be effective and one that might realistically be scaled up in the context of limited resources in a rural LMIC setting.

### Research questions

1. Which components of CBR are likely to be effective at improving functioning for people with schizophrenia?
2. Is CBR useful, acceptable and feasible from the perspective of people with schizophrenia and their caregivers?
3. What health service structures exist and how can they support CBR?
4. Is it possible to recruit, train and retain non-health workers to deliver CBR?
5. What community resources are available and are they accessible to people with schizophrenia?
6. Are community leaders willing and able to participate in CBR?
7. How can the positive effects of CBR be sustained?

## Methods

This study used a range of qualitative and participatory methodologies in five phases from September 2012 to March 2014. Intervention development was guided by a Theory of Change approach [35] in conjunction with the Medical Research Council framework for complex interventions [36]. The Theory of Change map provides a graphic representation of the causal pathways through which the RISE intervention is expected to achieve its impact [35]. The map includes (i) the **final outcome** (improved functioning in people with schizophrenia), (ii) **intermediate outcomes** that are needed to achieve the final outcome, (iii) **interventions** which are needed to move from one outcome to the next, (iv) **assumptions** (the conditions which the causal pathway needs in order to progress), (v) **rationale** for each link in the pathway and (vi) **indicators** (to evaluate whether each outcome is achieved). Assumptions articulated by the Theory of Change formed the research questions to be answered. We refined the map throughout the process as assumptions were tested and turned into rationale, or the intervention design was modified to fulfil assumptions.

### Ethics statement

The London School of Hygiene & Tropical Medicine Research Ethics Committee (reference 6408) and the Addis Ababa University College of Health Sciences Institutional Review Board (reference 039/13/PSY) granted ethical approval. Written informed consent was obtained from all participants in the in-depth interviews and focus group discussions. Only people with schizophrenia who had stable illness were invited to participate. Prior to conducting the interviews the psychiatrist assessed the individuals’ decision-making capacity. Only individuals judged to have decision-making capacity were included; these individuals continued to the consent process. Verbal informed consent was obtained from all participants in the Theory of Change workshops and recorded in the workshop notes. Individual written informed consent was not sought from workshop participants as the workshops were a participatory planning process; the workshops were not audio-recorded; vulnerable groups, i.e. people with schizophrenia or caregivers, were not included in the workshops; and the workshop outputs were two Theory of Change maps formed by group consensus (contributions were not attributed to individual participants).

### Phase 1: Identification of potential components of CBR for schizophrenia

#### Objectives

(i) Identify potential components of CBR and their likely effectiveness and (ii) describe how components could improve functioning.

#### Methods

A literature review of CBR for schizophrenia in LMIC was conducted. Other psychosocial interventions were also reviewed because CBR consists of many elements that have typically been evaluated separately. We reviewed resources from the WHO (CBR Guidelines) [17], COPSI [37], Rehabilitation And Prevention Initiative against Disabilities (RAPID) project, and other similar projects. RAPID is an Ethiopian CBR project for children with disabilities. RAPID is a local collaborator on RISE and is affiliated with CBM, an international disability and development organisation. Site visits and in-depth consultation with the RAPID management team were conducted. The collated information was used to create a draft Theory of Change map for how the CBR components could improve functioning in people with schizophrenia.

### Phase 2: Situational analysis

#### Objectives

(i) Describe the socio-demographic characteristics, health services and community resources of Sodo and (ii) describe the situation of people with schizophrenia in this context.

#### Methods

We drew on work conducted by the PRIME project in Sodo district, which has been described in detail elsewhere [33, 38–40]. In brief it involved (i) reviewing a situational analysis, which comprised public domain data relating to population, health and social indicators; mental health policies and plans; mental health treatment coverage and district level services [38] (ii) reviewing resource mapping data collected using a semi-structured instrument to systematically quantify community assets, for example traditional healers and religious groups and [39] (iii) reviewing qualitative data on the acceptability and feasibility of task-sharing mental health services [33, 40]. In addition rich local data relating to prevalence [41], clinical course and outcome [1, 7, 10], disability [2], mortality [6, 42], access to health services [10], beliefs [43, 44], caregiver burden [3, 45], experiences of stigma [4, 5, 9] and use of traditional healers [43] relating to schizophrenia was reviewed.

### Phase 3: Evaluation of the feasibility of CBR

#### Objectives

(i) Determine the feasibility of CBR components and delivery mechanisms and (ii) get local ‘buy-in’ for RISE.

#### Methods

The scoping workshop and first Theory of Change workshop involved eight experts in CBR and mental health. The second Theory of Change workshop included twenty community leaders from Sodo, including representatives of microfinance, *edir* (traditional burial association), education, police, traditional healers and religious leaders. Each workshop lasted half a day; the expert workshops were facilitated in English and the community workshop was facilitated in Amharic by an Ethiopian investigator (AF). At each workshop a large-scale (approximately 2 metres x 3 metres) draft Theory of Change map was presented to workshop participants and the various components explained. The map was refined in real time by investigators by adding notes on colour-coded paper and linking arrows. Photographs were taken of the map at the end of each workshop to maintain a visual record of the discussion. In addition, detailed minutes were taken in English by an Ethiopian research assistant. Following the workshops these visual and written records were combined to update the Theory of Change map.

### Phase 4: Evaluation of the acceptability of CBR

#### Objectives

(i) Describe unmet needs of people with schizophrenia and (ii) determine the acceptability of CBR.

#### Methods

We conducted 16 in-depth interviews and five focus group discussions (including 35 participants) with people with schizophrenia, caregivers, community and religious leaders, traditional healers, RAPID CBR workers, health extension workers and primary healthcare workers (see Table 1). Health extension workers are salaried community health workers engaged in health promotion and disease prevention. Participants were identified through the district health bureau (for staff), RAPID CBR project, and Butajira psychiatric outpatient clinic. Participants were purposively selected to ensure a spread of gender, work experience, type of community leader and functioning of people with schizophrenia. Topic guides covered key issues around acceptability and feasibility. The In-depth interviews and focus group discussions were conducted in Amharic by a male Ethiopian psychiatrist and a male Ethiopian PhD student (with a Psychology MSc). Both had experience in conducting interviews and discussion groups with people with schizophrenia and caregivers. The participants were contacted by telephone or face-to-face to invite them to participate; no potential participants refused to take part. The interviews were conducted at health centres and the research office in Butajira, a private room in Bui town, and the RAPID office in Adama. Participants were given information about the purpose of the study prior to the interviews, but no other relationship between the researchers and participants was established in advance. In-depth interviews lasted between 40 and 60 minutes and focus-group discussions lasted between 60 and 120 minutes and all were audio-recorded. The investigator conducting the main analysis (LA) observed all interviews and focus groups. Debrief discussions with the interviewers were held immediately afterwards; initial impressions and observations were captured in field notes. No repeat interviews or participant checking was carried out. The audio-recordings were transcribed in Amharic, and then translated into English. A framework analysis was conducted; this approach is recognised as suitable for intervention development work as interviews are typically structured with clear a priori themes [46]. NVivo for Mac software was used to manage the data. A coding scheme was developed using a priori core themes based on the topic guide (e.g. ideal characteristics of a CBR worker); and subsequently sub-themes and new themes that emerged through reading the manuscripts. Two investigators indexed two transcripts using the codes developed. Discrepancies between applications of the coding scheme were discussed and adjustments were made where required. One investigator then indexed all transcripts using the final coding scheme. A matrix was created charting data relating to each theme against each participant. Finally, themes were summarised and interpreted, noting associations between themes and patterns relating to participant characteristics (e.g. gender). Further interviews and focus groups were conducted until data saturation was reached.

**Table 1:** In-depth interviews and focus group discussion participants.

Stakeholder	Number of in-depth interviews	Number of focus group discussions
People with schizophrenia (male)	3	0
People with schizophrenia (female)	2	0
Caregivers (male)	1	1 (n = 8)
Caregivers (female)	1	1 (n = 7)
Community leaders (male)	7	0
Health extension workers	0	1 (n = 8)
Primary care workers	0	1 (n = 6)
CBR workers	2	1 (n = 6)
**Total**	**16**	**5 (n = 35)**

### Phase 5: Synthesis of results to finalise intervention

#### Objectives

(i) Finalise CBR content and delivery and (ii) develop CBR training materials.

#### Methods

**Fig 1:**
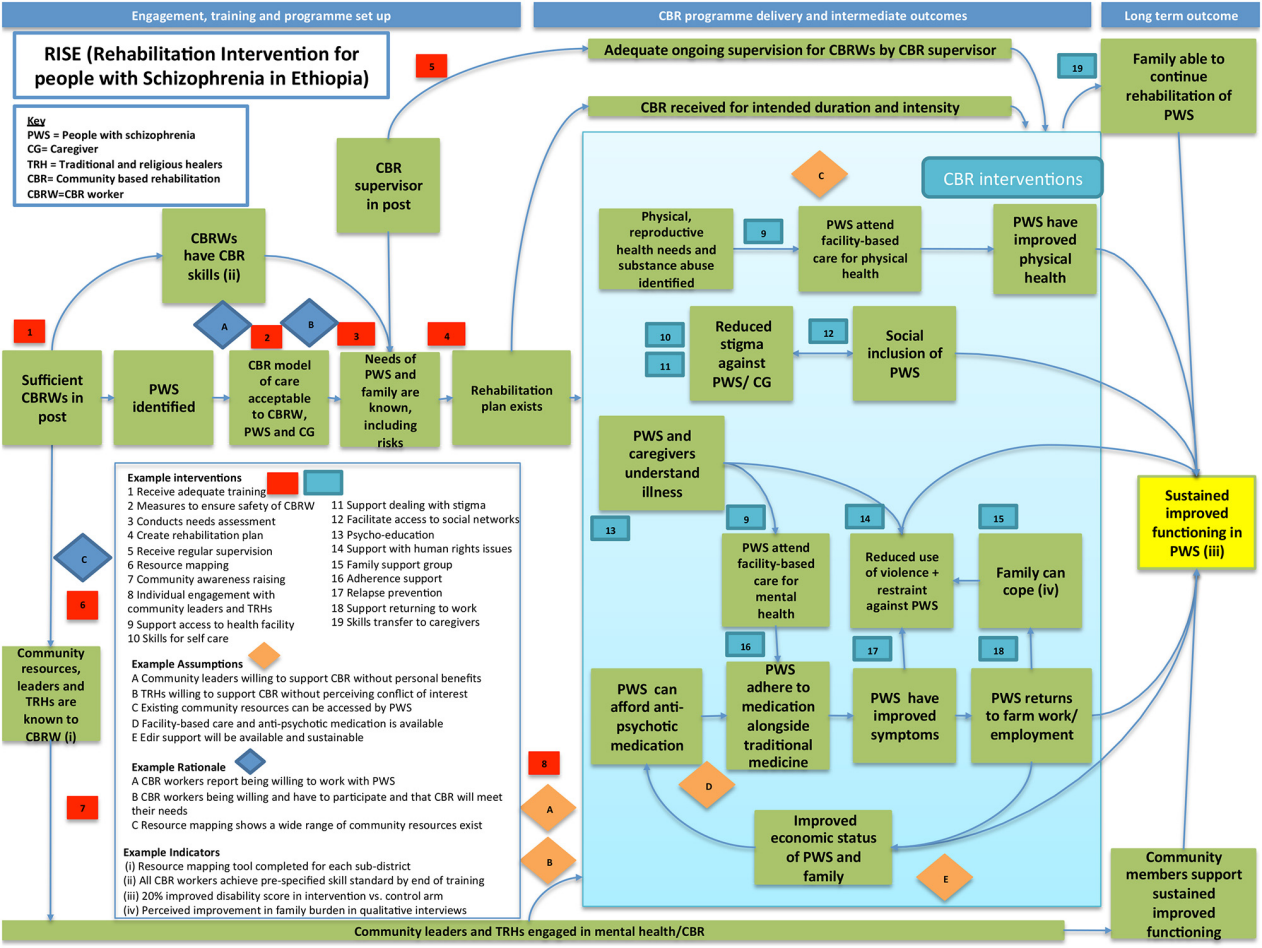
RISE Theory of Change map.

A three-day intervention-planning workshop involved Ethiopian psychiatrists, the CBM West Africa mental health advisor, CBM Ethiopia director, RAPID manager, and the senior health administrator for Sodo. The synthesised findings of Phases 1 to 4 were presented to participants, who decided the detailed content and structure of the intervention. The final Theory of Change map is presented in Fig 1.

## Results

The following section details how the research questions were answered using the different methods, and how these findings contributed to the final intervention (see S1 Table for details). Table 2 summarises the key themes generated from the in-depth interviews and focus group discussions (Phase 4).

**Table 2:** Summary of findings from in-depth interviews and focus group discussions.

Research question	Theme	Findings
**Which components of CBR are likely to be effective?**	*Necessity of community mobilisation element*	Community leaders have powerful influence on the community’s beliefs
		Community leaders are gatekeepers to community resources
**Is CBR useful, acceptable and feasible from the perspective of people with schizophrenia and caregivers?**	*Current problems and needs*	Family conflict
		Estranged from friends and neighbours
		Difficulty doing housework, farm work and business
		Problems with self care
		Stigma and discrimination
		Problems participating in community activities
		High caregiver burden
	*CBR content*	CBR perceived as acceptable and useful overall
		Caregivers and community leaders thought returning to work as central to regaining functional role and economic status; people with schizophrenia concerned about stress.
		Personal experiences of physical restraint or awareness of the practice amongst most participants; best approach to address this felt to be improved access to mental healthcare
	*CBR delivery*	Most participants preferred home visits
		Some participants preferred CBR workers the same gender as participants; others had no preference
		Desirable characteristics of CBR workers included being caring, understanding and knowledgeable, and having a good understanding of the local community
**Is it possible to recruit, train and retain field workers to deliver CBR?**	*Willingness of CBR workers*	Motivation to work with people with schizophrenia.
		Fears the work could be risky or stigmatizing
	*Ability of CBR workers*	Confident of ability to do work.
		Importance of field training, top-up training and peer supervision stressed
**What community resources are available in Sodo and are they accessible to people with schizophrenia?**	*Accessibility of community resources*	Problems accessing community resources due to stigma, problems with social interactions, lack of motivation and being symptomatic.
	*Role of edir (traditional burial association)*	Suggested role of *edir* included financial/material support, awareness raising, higher threshold for exclusion of people with schizophrenia when not contributing
**Are community leaders willing and able to participate in CBR?**	*Willingness of community*	Community leaders report sense of responsibility to support people with schizophrenia.
		Caregivers sceptical that support will be available.
	*Willingness of traditional or religious healers to participate*	Healers reported willingness to signpost to health centre/medication.
		Some reports of healers warning against medication use. Mixed views as to whether healers would receive education and change practices.
	*Willingness to work with either gender*	Community leaders state they are willing to work with CBR workers of either gender- skills are more important than gender

### 1. Which components of CBR are likely to be effective at improving functioning for people with schizophrenia?

Potential components of CBR for schizophrenia were listed according to the CBR pillars (health, social, livelihood, empowerment and education- see Table 3); whilst many relate to health, other pillars were also represented. The strongest evidence related to psycho-education [47–52], family intervention [23, 24, 53, 54] and adherence support [55, 56]. Multi-component interventions were effective, but it was difficult to elucidate their ‘active ingredient/s’ from the results [19, 20, 26]. We took into account whether potential CBR components were likely to be effective for the ‘difficult to treat’ group who will participate in the RISE trial. Novel components of CBR for schizophrenia included family intervention, stress and anger management and dealing with distressing symptoms (e.g. hallucinations and delusions). A schema of how CBR components could improve functioning was incorporated into the Theory of Change map (Fig 1). Many components may act in synergy, and also through intermediate outcomes and positive feedback loops. For example rehabilitation focused on returning to farm work, as well as directly improving functioning, may improve the ability of individuals to pay for medication, which in turn improves symptoms and therefore functioning. Support with medication adherence is also likely to improve symptoms; together with a family intervention this will reduce the need for physical restraint, which would also lead to return to previous functional roles.

**Table 3:** Potential components of community-based rehabilitation for schizophrenia.

HEALTH	SOCIAL	LIVELIHOOD	EMPOWERMENT	EDUCATION
Psycho-education ++	Support with self-care +	Facilitating access to social protection +	Addressing human rights -	Facilitating access to adult education -
Adherence support ++	Social skills training +	Supporting return to work +	Individual stigma reduction strategies -	
Family intervention ++	Supporting return to social activities +	Facilitating access to microfinance +	Self-help initiatives +	
Relapse prevention plan +		Mobilising community support +	Community-awareness raising -	
Support for distressing symptoms +				
Support accessing health services +				
Stress and anger management +				

Strength of evidence in literature review:

(++) Strong evidence

(+) Weak evidence/ part of multi-component study

(-) Insufficient studies.

There was strong support for community mobilisation from all stakeholders. Disability arises due to both illness and societal factors [57] so an intervention addressing both elements is indicated. There was consensus that community leaders have a powerful influence on the views of the community and are gatekeepers to community resources needed for CBR (“*The community won’t believe in things that the leaders don’t believe in”* (FGD 03, health extension worker)). This was an important finding given the strong influence of stigma on the experiences and social functioning of people with schizophrenia. Structured community mobilisation was therefore prioritised to maximise the effectiveness and sustainability of CBR. As shown on the theory of change map, identification and mobilisation of community resources are intermediate outcomes that are necessary foundations for ensuring the sustainability of the family level interventions.

### 2. Is CBR useful, acceptable and feasible from the perspective of people with schizophrenia and caregivers?

People with schizophrenia and caregivers were found to have a range of unmet needs and problems. Issues included conflict within families, being estranged from friends, difficulty doing housework, farm work and business and problems with self-care. Many participants with schizophrenia and caregivers reported experiences of stigma and other types of participants were aware it was a common occurrence. Instances included being called names, being laughed at or gossiped about, losing friends, being discouraged from participating in social life, and not being trusted in the workplace or in other settings. One male caregiver reported, “*No institution*, *no organization invites persons with mental illness to participate… because they are people with problems*, *saying that they will ruin things… they will not perform the work properly"* (FGD 01, male caregiver). Another caregiver explained, “*They won’t accept their word*. *Even if he [her son with schizophrenia] speaks the truth*, *they would say*, *‘he is a patient*, *don’t say anything back’*. *He is a patient*. *Now they say*, *‘is he possessed by the devil*?*’”* (FGD 02, female caregiver). Problems with participating in conventional community activities, for example drinking coffee with family and neighbours, attending church or mosque and attending weddings and funerals, were also reported. Participants named a variety of reasons for these problems, including side effects of medication, being unwell or unmotivated, having poor social skills, and stigmatising attitudes of community members. The range of needs highlighted the requirement for detailed needs assessments for participants. CBR would then be tailored accordingly, as opposed to a ‘one size fits all’ approach. Focusing on the participants’ expressed needs was perceived to be an important approach for maintaining engagement in the programme.

Caregivers were found to have high burden relating to financial problems (often due to costs of treatment), fear for personal safety, stigma and problems with social life. One caregiver said, “*We hide knives and tools from him because we are scared of him… Just in case he got upset all of a sudden…He might kill someone or he might destroy or burn someone’s property*” (IV02, female caregiver). Several reported that they had become ill through caring: “*We all became sick because of him*” (IV03, male caregiver). In light of this, guidance on assessing and addressing caregiver problems was added to the intervention.

All stakeholder groups viewed CBR as an acceptable and useful approach. First, because medication alone did not solve all problems; psychosocial support and rehabilitation were also needed. Second, because external ‘expert’ advice was likely to have greater influence on individuals with schizophrenia than advice from family members. Third, because an individual’s recovery could benefit the whole community, particularly if they could return to work. Finally, CBR was seen as empowering (“*[CBR] is essential for people with mental illness…*.*to live on equal bases in terms of ways of thinking and attitude”* (FGD 01, male caregiver).

For many participants, support returning to work was crucial for improving functioning, economic status and reducing family burden. However there were mixed views from people with schizophrenia. One participant was keen to receive a business loan, whilst two others only wanted simple work, and found dealing with money stressful. Most people in Sodo are subsistence farmers, there is limited formal employment and no vocational rehabilitation facilities. Accordingly, it was decided that vocational rehabilitation, whilst important, would typically focus on developing skills required for returning to farm work or daily labouring. Due to concerns that microfinance (such as cooperative savings and loans schemes) may increase stress in participants [58, 59] or may result in exploitation, it was decided to exclude a microfinance component from the RISE intervention. There was also particular backing for psycho-education, family support and support with adherence and accessing health services. Furthermore, the qualitative findings indicated that specific components were needed to address problems with day to day functioning (for example dressing independently) and participation in community activities (to include support with regaining specific social skills).

Some participants perceived self-help groups as useful for support and information sharing. There were examples of female caregivers and people with schizophrenia already meeting to discuss their problems. Together these findings suggested self-help initiatives could be a useful component, even without a savings and loans element.

Most participants were aware of physical restraint of people with schizophrenia, with some speaking from personal experience. The reasons for restraint included protecting the individual (from themselves or others), protection of others, as a means to transport the person to the health centre, and as a means to force the individual to take medication.

Consensus across stakeholder groups was that the best way to reduce chaining was to increase access to treatment. Community leaders felt the family should be educated about the harmful effects of chaining. There was concern amongst community leaders that unchaining may put other community members at risk. The decision was made to focus on avoiding chaining through access to treatment. The CBR worker should not instigate unchaining, but should work with the supervisor and health centre to ensure unchaining happens safely. In addition we would include pragmatic advice on how to restrain in a safe and dignified way, when it was needed as a last resort.

There are 49 herbalists, 21 *tanqway* (‘sorcerers’) and 27 holy water sites across the district [39]. Holy water, which is used for bathing or drinking at sites associated with the Orthodox Church, is believed to have curative properties. Holy water use reflects a prevailing belief in Ethiopian culture that severe mental illness is attributable to supernatural forces, such as possession by spirits or the shadow cast by the ‘evil eye’ [43, 44, 60]. The use of holy water is sanctioned by the dominant religious authorities. A visit to a holy water site may last from days to months. Whilst the Orthodox priests based at these holy water sites may provide spiritual guidance to people with mental illness, they do not typically take on an active caring role with individuals. However attendants based at holy water sites often house and feed holy water attenders for a fee paid by the family. In the current study holy water was used by several people with schizophrenia, often alongside taking medication. Primary care workers and community leaders also perceived *tanqway* to be commonly used. Previous research showed that 37% of people with schizophrenia in this area attend a traditional healer [7] and 30.9% of those obtaining treatment at Ammanuel Psychiatric Hospital in Addis Ababa had first sought help from priests or holy water sites [61]. There were experiences of medication being both encouraged and discouraged by holy water priests, and a few experiences of being restrained or beaten for refusing to take holy water. Education about the risks and benefits of holy water and traditional healing was included, with the focus on encouraging the use of medication alongside traditional treatments.

The high levels of poverty and long distances to roads, health centres and public transport meant participants would have difficulty travelling for CBR. The majority therefore wanted home visits, rather than visiting the health centre. There were no concerns about increased stigma resulting from home visits. Two people with schizophrenia who preferred CBR visits at the health facility wanted to be more active. CBR would therefore be delivered at home as standard, with health facility visits offered as an alternative in order to maximise engagement. People with schizophrenia and caregivers reported they could be flexible about the timing of visit. Nevertheless the importance of fitting around the participants’ schedules was emphasised, in order to minimise dropouts from the intervention.

Several caregivers and community leaders felt that the gender of the CBR workers did not matter; it was their skills that would be important. Others felt that CBR workers should be the same gender as their clients, as this would improve their relationship. It was decided that CBR workers of both genders would be recruited, whilst acknowledging that gendered allocation of CBR workers to participants would not be possible due to the cluster design of the RISE trial.

Desirable characteristics of CBR workers included being caring, understanding and knowledgeable, and having a good understanding of the local community. CBR workers would therefore be high school completers recruited from the immediate area. CBR worker competence evaluation would include communication skills.

### 3. What health service structures exist and how can they support CBR?

Primary care is delivered through eight health centres, staffed by health officers and nurses. The district’s first hospital is under construction. Most kebeles (sub-districts) are covered by one or two health extension workers. Health care costs are largely out-of-pocket with a free waiver available for the very poorest [38]. In mid-2014 primary care staff were trained in mental health diagnosis and treatment by PRIME. The health extension workers initially represented a potential workforce to deliver CBR and the health centre staff represented potential collaborators. However it was ultimately decided CBR should be delivered by CBR workers, recruited and trained specifically for RISE, rather than health extension workers. The rationale was that HEWs would not have time to deliver CBR on top of their usual duties. In addition there were five kebeles without a health extension worker and concerns about further drop-outs. There was consensus that CBR should be linked to health centres but that primary care staff would have minimal capacity to support rehabilitation. Two RISE supervisors will cover eight CBR workers, using one-to-one supervision, group supervision and unannounced observed home visits.

### 4. Is it possible to recruit, train and retain non-health workers to deliver CBR?

Community leaders and experts predicted difficulty recruiting CBR workers willing to work with people with schizophrenia. A minority were concerned that CBR workers would not be able to provide psychosocial support. However, CBR workers (for physical disabilities), and health extension workers were motivated to do the work, stating it would be rewarding and clearly needed. Yet there were also fears that the work could be dangerous or stigmatizing for CBR workers. These concerns underlined that adequate safety procedures for CBR workers were an essential intervention for CBR to succeed (including risk assessment, provision of mobile phones, training to deal with challenging situations, and, where a risk is identified, joint visits with the supervisor), whilst conveying a balanced sense of the risks associated with working with people with schizophrenia. It was decided to employ at least one male supervisor to maximise the safety of CBR workers.

Health extension workers and CBR workers were confident that with training they could undertake CBR work for schizophrenia, emphasising that the core skills of CBR workers are generic. They stressed the importance of practical training, shadowing existing CBR workers, and top-up training. Both one-to-one and group supervision were recommended, with the latter being particularly helpful for overcoming difficulties. The need for physically fit CBR workers was highlighted as there were no resources for car travel. Ability to walk long distances was therefore included in the recruitment criteria.

### 5. What community resources are available and accessible to people with schizophrenia?

Each kebele in Sodo district has churches, religious groups, women and youth associations. 33 kebeles have a government literacy programme, and most have a government microfinance initiative. There are no mental health users groups or disabled people’s organizations [39]. The rich community resources suggested that CBR workers should do resource mapping when they first start work in a kebele. There were mixed views as to whether people with schizophrenia have problems accessing existing community resources. All kebeles have several *edir*. Although *edir* is ostensibly a burial association, there was agreement that it is “*the most important community mobilizing agent*.*" (IV08*, *edir leader)* and should be engaged as a conduit for social inclusion and stigma reduction. Possible examples of community mobilisation were identified, including church leaders vocalising public support for an individual or assisting the family to take them to the health facility; and *edir* groups or wealthy individuals mobilising funds from community members to provide food or shelter for people with mental illness. However there were mixed views about *edir’s* potential role as a provider of social protection or material support. The intervention was therefore modified to indicate that financial support from *edir* should arise organically and not be demanded by the CBR worker.

### 6. Are community leaders willing and able to participate in CBR?

There were conflicting views as to whether community leaders would use their authority to support CBR without personal benefits. This was recognised as a key assumption to fulfil in order for the intervention to succeed. Female caregivers, based on their previous experiences, were sceptical that community leaders would provide support, whilst community leaders themselves described a sense of responsibility and were keen to collaborate. The importance of first raising awareness amongst community leaders was highlighted, with emphasis to be made on the benefits for the whole community of an individual’s recovery. The success of community mobilisation would then be reliant on community members taking ownership of the issue and identifying for themselves the ways they could help people with schizophrenia. There were mixed views as to whether holy water priests would be receptive to education about schizophrenia and the extent to which this would change their practices. It was decided that engagement with holy water priests and traditional healers would be instigated only where the family perceived this as useful. Concerns were raised as to whether community leaders would engage with female CBR workers, but community leaders themselves reported that skills were more important than gender.

### 7. How can the positive effects of CBR be sustained?

RAPID uses a combination of skills transfer to caregivers, parent groups and CBR committees (consisting of leaders from a range of sectors) to ensure the positive effects of CBR continue once the CBR workers had left the area. As it would be unfeasible to create new structures to ensure sustainability, it was decided that *edir* groups should be encouraged to take ownership of CBR. Family support groups were incorporated in the intervention and skills transfer to caregivers was highlighted as a key principle of CBR.

### Summary of final RISE intervention

**Fig 2:**
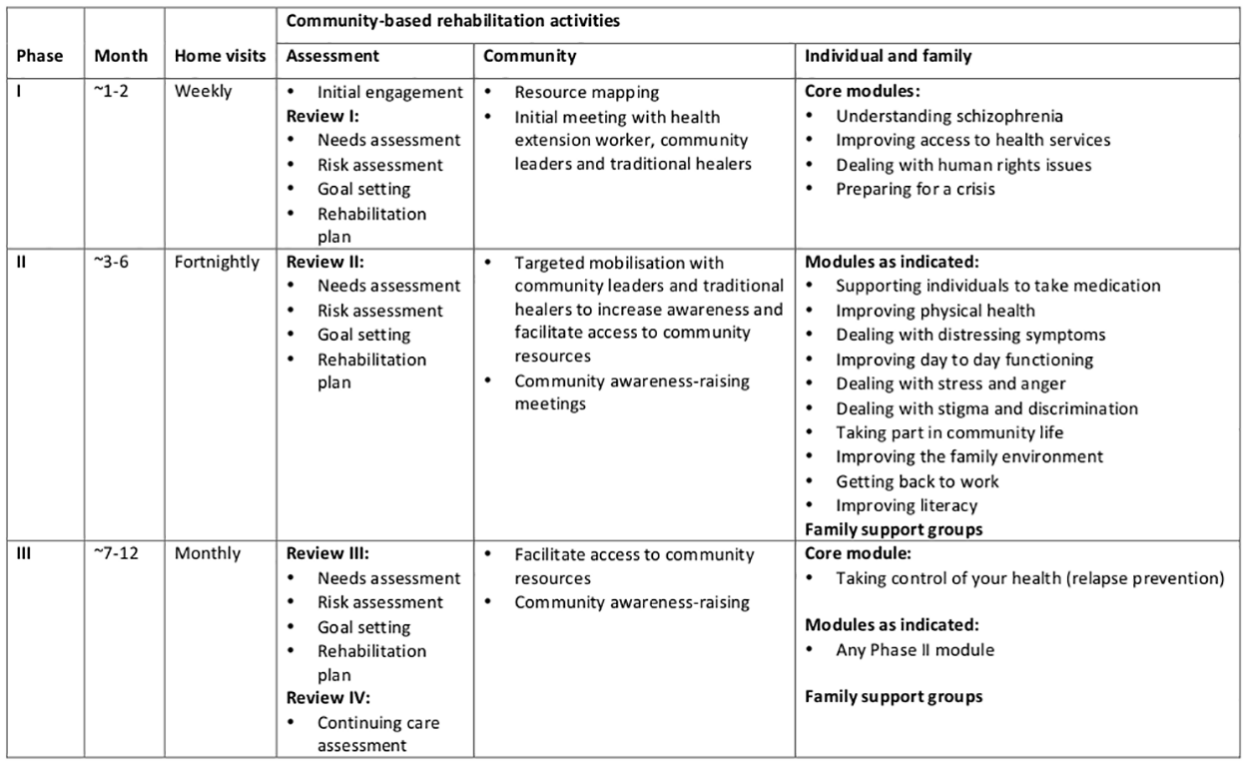
Overview of RISE intervention structure

The focus of the RISE CBR intervention will be on the individual developing the skills and confidence to perform their previous or desired roles and activities. These may relate to family life, work and community life. The intervention will be recovery oriented, emphasising hope and the individual’s strengths. Fig 2 summarises the final intervention structure. Basic counselling and problem solving skills will be employed by CBR workers to deliver the intervention. The intervention is delivered in three phases. In Phase 1, lasting one to two months, there are weekly home visits and the focus is on engagement with the family and addressing core needs through compulsory modules such as “Understanding Schizophrenia”. Following a needs and risk assessment, structured goal setting will be used to support individuals to select appropriate goals from a pre-defined list. In addition to four core modules, the goal selection will determine which additional CBR components the individual will receive. In Phase 2, lasting approximately five to six months, home visits are every two weeks and address the specific needs of the individual through optional modules such as “Getting Back to Work”. In Phase 3, lasting approximately four months, the emphasis is on preventing relapse as well as maintaining the progress made towards addressing specific needs. The three intervention phases reflect the changing needs of participants over 12 months. The transition between phases is conditional on achievement of goals rather than specific time points.

Community mobilisation work will run alongside family-based components. Community mobilisation involves identifying local community resources and leaders, awareness-raising meetings at existing community groups (for example women’s saving groups) and targeted meetings with community leaders addressing specific needs of participants (for example identifying sources of food or financial support, or encouraging patients to attend the health centre). Whilst support with medication adherence is an important component, CBR workers will not prescribe or deliver medication. Indeed participants who are unable or unwilling to take anti-psychotic medication will nevertheless continue CBR and be supported to achieve goals related to functioning, which is the primary aim of the intervention. The five-week training programme for CBR workers will include 50% practical and 50% fieldwork, and will follow a training manual adapted from the COPSI manual.

## Discussion

This study is a systematic and theory-driven effort to design a CBR intervention for schizophrenia in a resource-poor setting. This preparatory work aimed at designing the RISE intervention is an innovative attempt to tailor CBR’s capacity to promote inclusion and improve access to essential services to the needs of people with schizophrenia. In doing so it aims to bridge the gap between health services and a more community-oriented development model of disability [34]. Whilst integration of mental health into existing CBR programmes has typically involved in-depth consultation work, this has not usually been theory-driven. The RISE CBR intervention has important differences compared to other models of community care for schizophrenia in LMIC. First, distinct from other interventions, such as COPSI [26] and a South African ACT programme [22], there is a substantial community mobilisation element. Our results indicate that community participation is likely to be essential for improving social inclusion, as well as having a role in improving medication adherence, reducing experiences of stigma and improving economic status. Utilising the CBR model, in which community mobilisation is key, therefore represents a major strength of the RISE intervention. Second, there is no collaboration with mental health specialists [30, 33]. This reflects the reality in this setting that the majority of care for people with schizophrenia is delivered by primary care staff, who have themselves only recently been trained in mental health. Third, there is a more structured approach to setting goals for individuals and selecting intervention components compared to other interventions [26].

We compromised on immediate scalability to construct a feasible intervention. The use of specialist CBR workers instead of existing health extension workers means that additional resources are required to scale up CBR, and as such RISE is a proof of concept study. This was a pragmatic approach given the widespread recognition that scaling up of mental healthcare cannot be done without extra resources. The RISE intervention is designed to be scalable with limited resources, for example only people with schizophrenia who are still unwell or disabled after six months access to facility-based care are included. The RISE trial will determine whether this intervention is cost-effective and therefore a potentially suitable investment for governments and other funders including the Ethiopian Ministry of Health. The collaboration between the Ministry of Health and PRIME (in which RISE is nested) could potentially pave the way for the scale up of CBR for schizophrenia. Furthermore, by utilising CBR workers (rather than existing health workers) and collaborating with CBM we have designed an intervention that meets the needs of, and is compatible with, CBR projects for other disabilities. This will provide evidence for integration of mental health into the large network of existing CBR projects run by NGOs.

This study addresses a criticism of global mental health research by developing a socially and culturally relevant psychosocial intervention using participatory methods [62]. The Theory of Change approach allows assumptions and barriers to be articulated and tested using a range of research methods. The Theory of Change map gives a visual record of modifications to the intervention on the basis of the research findings. A set of indicators has been developed, including trial outcomes and process data. This gives us a theoretical framework that we can later use to identify which are the most important components of CBR as part of the formal evaluation in an RCT, allowing us to refine an effective intervention for scaling up.

There may have been social desirability bias, particularly from community leaders, as there may be political pressure to express support of government initiatives. As PRIME, in which RISE is nested, is a collaboration with the Ethiopian Ministry of Health, community leaders may have felt compelled to champion CBR. This could explain their different opinion regarding community support compared to female caregivers, who are not susceptible to the same pressures. As the investigators are invested in the RISE project it is possible this led to a biased interpretation of the qualitative data, emphasising a favourable opinion of CBR amongst participants. While no meaningful ownership of the Theory of Change map can be claimed by stakeholders outside of the workshops, those who participated represented relevant stakeholders, and Theory of Change was undoubtedly a useful tool throughout the process.

## Conclusion

Extensive formative research using a variety of methods nested within a Theory of Change framework has enabled the design of a culturally appropriate complex mental health intervention that is acceptable and feasible to service users and providers. This CBR intervention will be finalised in a pilot and then tested in a cluster-randomised trial to determine its effectiveness and cost-effectiveness in improving functioning in people with schizophrenia in a rural district in Ethiopia.

## Supporting Information

S1 Table Summary of research questions, findings and impact on intervention design. (DOCX)

## Abbreviations

CBR: Community-based rehabilitation
COPSI: COmmunity care for People with Schizophrenia in India
LMIC: Low and middle Income countries
NGO: Non governmental organisation
PRIME: PRogramme for Improving Mental healthcarE
RAPID: Rehabilitation And Prevention Initiative against Disabilities
RCT: Randomised controlled trial
RISE: Rehabilitation Intervention for people with Schizophrenia in Ethiopia
WHO: World Health Organisation
