# Supplementary material for: Development of a Community-Based Rehabilitation Intervention for People with Schizophrenia in Ethiopia
Source: PLoS One. 2015 Nov 30;10(11):e0143572. doi: 10.1371/journal.pone.0143572 (PMC4664267; doi:10.1371/journal.pone.0143572)
Supplement: S1 Table — (DOCX) [file pone.0143572.s001.docx]

S1 Table: Summary of research questions, findings and impact on intervention design

| **Research questions and assumption** | **Phase tested** | **Finding** | **Impact on intervention design** |
| --- | --- | --- | --- |
| **1. Which potential components of CBR are likely to be effective at improving functioning for people with schizophrenia?** | | | |
| **CBR can improve functioning in people with schizophrenia** | 1 | Good evidence that community care, psycho-education, adherence support, and family intervention can improve functioning | - Include broad range of CBR components, which may contribute directly or indirectly to improved functioning. |
|  | 1, 3, 4 | Some CBR components for which there is no research evidence could contribute to improved functioning via intermediate outcomes |  |
| **A community engagement approach is required in addition to family-based care** | 1 | Community engagement is advocated by WHO CBR guidelines and has been shown in observational studies in India to improve functioning | - Incorporate community engagement, targeting leaders, to maximise effectiveness of family-based components and sustainability. |
|  | 3, 4 | Community leaders have powerful influence on views and behaviour of community and may be gatekeepers to community resources. |  |
| **2. Is CBR useful, acceptable and feasible from the perspective of people with schizophrenia and their caregivers?** | | | |
| **CBR can address the needs of people with schizophrenia** | 2 | High levels of disability, stigma and family burden. Most work as subsistence farmers. Few formal employment opportunities; no vocational rehabilitation facilities. | - Common needs will be addressed in core modules. Modules addressing other needs should be delivered if indicated by needs assessment and goal setting. - Include guidance on addressing caregivers needs - Vocational rehabilitation to focus on returning to farm work. |
|  | 3 | Needs assessment and rehabilitation plan are essential. Return to work perceived as key step for improving functioning, economic status and reducing family burden. |  |
|  | 4 | People with schizophrenia and caregivers have diverse and varied needs. CBR components perceived to be useful for addressing needs. |  |
| **People with schizophrenia and their caregivers are willing and have time to participate in CBR** | 4 | Near universal willingness and enthusiasm to participate. General flexibility and no concerns about not having time.  CBR workers should be from local area, have a caring attitude and be knowledgeable. Mixed views on preferred gender. | - Recruit CBRWs from local area of both genders. - Incorporate training on empathy and building trust. |
| **The benefits of microfinance outweigh the risks** | 1 | Some evidence that microfinance can increase stress | - Facilitating inclusion in existing microfinance schemes excluded from CBR |
|  | 3 | Concerns about abuse or exploitation of people with schizophrenia |  |
|  | 4 | Microfinance may be unacceptable to some participants |  |
| **Self-help initiatives are perceived to be beneficial** | 4 | Self-help groups perceived to be useful for moral support and information sharing. | - Family support groups included in CBR. |
| **People with schizophrenia are commonly chained or restrained** | 3 | Chaining may be a result of stigma and low awareness. Community leaders could help to reduce chaining by raising awareness. | - CBR should address chaining but focus should be on accessing treatment. - CBR workers should not initiate unchaining but should refer to health centre and supervisor - CBR workers should educate families on how to chain safely. |
|  | 4 | Chaining is common at home and at holy water sites. The best way to reduce it is to improve access to treatment. Community leaders concerned about safety issues related to unchaining. |  |
| **TRHs are typically used as a source of care for people with schizophrenia** | 2 | 49 herbalists, 21 *tanqway* (‘witch doctors’) and 27 holy water sites across the district. 37.4% of people with schizophrenia attend a traditional healer | - Educate family about risks and benefits of TRH. Do not forbid, but encourage use alongside medication. |
|  | 3 | Holy water use is common, often alongside medication. |  |
|  | 4 | Should aim to encourage holy water use alongside medication. |  |
| **3.What health service structures exist and how can they be utilised to support delivery of CBR?** | | | |
| **Health extension workers have the capacity to deliver CBR** | 2 | HEWs not present in every kebele | - Recruit and train a new cadre of worker, CBR workers, especially for this project |
|  | 3 | Consensus that HEWs would not have the capacity to deliver CBR on top of current work; in addition high rates of drop out from HEW posts. |  |
| **Health centre staff have the capacity to support CBR** | 2 | Primary care staff newly trained to diagnose and treat schizophrenia | - Supervision internal to RISE rather than from the health centre. - Monitor health centre attendance |
|  | 3 | CBR should be linked to health centres but primary care staff will have minimal capacity to support rehabilitation. CBR supervisors should supervise CBR workers. |  |
| **Medication is available at the health centre** | 2 | PRIME will ensure continuous medication supply to health centres | - CBRW can facilitate free medication certificate. |
|  | 3 | Free medication certificate available for minority from kebele official |  |
| **4. Is it possible to recruit, train and retain field workers to deliver CBR?** | | | |
| **CBR workers are willing to work with people with schizophrenia** | 3 | Concerns potential CBR worker recruits may be afraid to do home visits to people with schizophrenia. Also concerns about drop out. | - Comprehensive safety procedures for CBR workers - Training to give a balanced sense of risk - Male and female supervisors. |
|  | 4 | CBR workers, HEWs and primary care staff motivated to work with people with schizophrenia. But fears the work could be risky or stigmatizing |  |
| **CBR workers will have adequate CBR skills** | 1 | Lay people previously trained as CBR workers in India and RAPID | - Training to including shadowing existing CBR workers and top-up training. - Training to include problem solving and communication skills. - 1:4 ratio of supervisors to CBR workers. One-to-one sessions and group supervision. |
|  | 3 | Concerns about ability to train non-specialists to deliver CBR for schizophrenia. Importance of supervisors doing ongoing assessment of gaps in skills/knowledge. Don’t need formal counselling training. |  |
|  | 4 | HEWS and CBR workers confident they could do CBR for schizophrenia. Importance of field training, top-up training and peer supervision |  |
| **CBR workers can overcome logistical issues to deliver CBR** | 2 | Mainly rural, many kebeles remote from towns, roads, public transport | - Include willingness/ability to walk long distances in recruitment criteria |
|  | 3,4 | CBR workers need to be strong, fit and capable of walking long distances |  |
| **5. What community resources are available in Sodo and are they accessible to people with schizophrenia?** | | | |
| **Existing community resources are available** | 2 | Rich community resources e.g. microfinance and literacy schemes, Women’s and youth associations, churches, mosques and informal religious groups, and Edir groups (traditional burial association) | - CBR workers to conduct resource mapping when they start working in a kebele. - Potential targets for social inclusion identified e.g. edir, mahaber |
|  | 3 | Agreement that *edir* is a powerful social structure. Confirmation of presence and activity of other community resources. |  |
| **Existing community resources can be accessed by people with schizophrenia** | 3 | Mixed views on whether people with schizophrenia have problems accessing community resources. May be opportunities for income generation | - Assess for daily labouring opportunities. - CBR worker to facilitate access to community resources May need to liaise with relevant community/religious leaders. - Support with social skills to be included. |
|  | 4 | Problems accessing community resources due to stigma, problems with social interactions, lack of motivation and being symptomatic. |  |
| **Edir support will be available and sustainable** | 3 | Mixed views about edir’s role as a provider of social protection or material support. New unions previously formed to support people with HIV- suggested this could be replicated for people with schizophrenia. | - More feasible to get support from existing structures i.e. Edir, than to encourage formation of new structures. - support from Edir should arise organically, not be expected by CBR worker. |
|  | 4 | Suggested role of Edir: financial/material support, awareness raising, higher threshold for exclusion of people with schizophrenia when not contributing |  |
| **6. Are community leaders willing and able to participate in CBR?** | | | |
| **Community leaders will participate in CBR without personal benefits** | 3 | Concerns that community leaders will not participate in CBR | - Include community awareness raising and engagement with community leaders at start of intervention. |
|  | 4 | Community leaders report sense of responsibility to support people with schizophrenia. Caregivers sceptical support will be available. |  |
| **Traditional and religious healers will participate in CBR without perceiving a conflict of interest** | 3 | TRHs and CBR workers would need joint understanding of person with schizophrenia’s needs. TRH may not be willing to discuss individuals. | - Engagement with TRHs to be instigated where the individual and family perceived this as a useful adjunct to family support and general community awareness-raising. |
|  | 4 | TRHs states willingness to signpost to health centre/medication. Some reports of TRHs warning against medication use. Mixed views as to whether TRHS would receive education and change practices. |  |
| **Community leaders will be willing to work with male and female CBR workers** | 3 | Community leaders may only engage with male CBR workers | - Recruit on basis of interest and post-training competence, not gender |
|  | 4 | Community leaders state they are willing to work with CBR workers of either gender- skills are more important than gender |  |
| **7. How can the positive effects of CBR be sustained?** | | | |
| **CBR can continue after the CBR worker has left** | 1 | Issues with sustainability identified in previous study | - Skills transfer to caregiver. - Focus on Edir to ensure sustainability CBR committee not feasible. |
|  | 3 | RAPID uses CBR committees to ensure sustainability. Transfer of skills to caregiver also essential. |  |

CBR= Community-based rehabilitation; RAPID= Rehabilitation And Prevention Initiative against Disabilities Project; Edir= traditional burial association; ; HEW= Health extension worker; TRH= traditional and religious healer; PRIME= PRogramme for Improving Mental healthcare; kebele= sub-district
